# Prognostic Value of Baseline Radiomic Features of ^18^F-FDG PET in Patients with Diffuse Large B-Cell Lymphoma

**DOI:** 10.3390/diagnostics11010036

**Published:** 2020-12-28

**Authors:** Kun-Han Lue, Yi-Feng Wu, Hsin-Hon Lin, Tsung-Cheng Hsieh, Shu-Hsin Liu, Sheng-Chieh Chan, Yu-Hung Chen

**Affiliations:** 1 Department of Medical Imaging and Radiological Sciences, Tzu Chi University of Science and Technology, Hualien 97005, Taiwan; john.lue@protonmail.com (K.-H.L.); kaopectin@yahoo.com.tw (S.-H.L.); 2 Department of Hematology and Oncology, Hualien Tzu Chi Hospital, Buddhist Tzu Chi Medical Foundation, Hualien 97004, Taiwan; wuyifeng43@gmail.com; 3 Department of Medicine, College of Medicine, Tzu Chi University, Hualien 97004, Taiwan; williamsm.tw@gmail.com; 4 Medical Physics Research Center, Institute for Radiological Research, Chang Gung University/Chang Gung Memorial Hospital, Taoyuan 33302, Taiwan; muska0345@outlook.com; 5 Department of Radiation Oncology, Chang Gung Memorial Hospital, Taoyuan 33305, Taiwan; 6 Department of Nuclear Medicine, Keelung Chang Gung Memorial Hospital, Keelung 20401, Taiwan; 7 Institute of Medical Sciences, Tzu Chi University, Hualien 97004, Taiwan; tchsieh@gms.tcu.edu.tw; 8 Department of Nuclear Medicine, Hualien Tzu Chi Hospital, Buddhist Tzu Chi Medical Foundation, Hualien 97004, Taiwan

**Keywords:** ^18^F-FDG, PET, radiomics, prognosis, diffuse large B-cell lymphoma

## Abstract

This study investigates whether baseline ^18^F-FDG PET radiomic features can predict survival outcomes in patients with diffuse large B-cell lymphoma (DLBCL). We retrospectively enrolled 83 patients diagnosed with DLBCL who underwent ^18^F-FDG PET scans before treatment. The patients were divided into the training cohort (*n* = 58) and the validation cohort (*n* = 25). Eighty radiomic features were extracted from the PET images for each patient. Least absolute shrinkage and selection operator regression were used to reduce the dimensionality within radiomic features. Cox proportional hazards model was used to determine the prognostic factors for progression-free survival (PFS) and overall survival (OS). A prognostic stratification model was built in the training cohort and validated in the validation cohort using Kaplan–Meier survival analysis. In the training cohort, run length non-uniformity (RLN), extracted from a gray level run length matrix (GLRLM), was independently associated with PFS (hazard ratio (HR) = 15.7, *p* = 0.007) and OS (HR = 8.64, *p* = 0.040). The International Prognostic Index was an independent prognostic factor for OS (HR = 2.63, *p* = 0.049). A prognostic stratification model was devised based on both risk factors, which allowed identification of three risk groups for PFS and OS in the training (*p* < 0.001 and *p* < 0.001) and validation (*p* < 0.001 and *p* = 0.020) cohorts. Our results indicate that the baseline ^18^F-FDG PET radiomic feature, RLN_GLRLM_, is an independent prognostic factor for survival outcomes. Furthermore, we propose a prognostic stratification model that may enable tailored therapeutic strategies for patients with DLBCL.

## 1. Introduction

Diffuse large B-cell lymphoma (DLBCL) is the most common type of lymphoma, accounting for approximately one-third of non-Hodgkin lymphomas [1]. DLBCL is a heterogeneous group of lymphomas with variable survival rates. The cure rate of DLBCL has improved substantially due to advances in disease management, and the addition of rituximab immunotherapy to conventional cyclophosphamide, hydroxydaunorubicin (doxorubicin or epirubicin), oncovin (vincristine), and prednisolone chemotherapy (R-CHOP) is effective in 60–70% of patients [2]. However, approximately 30–40% of patients still suffer relapse or refectory disease [3]. New prognostic factors for personalized risk-adapted treatment is currently an unmet clinical need, and may improve the outcomes of patients with DLBCL.

The International Prognostic Index (IPI) has been the basis for determining prognosis for DLBLC in clinical practice for the past 20 years [4,5]. In addition to IPI, ^18^F-fluorodeoxyglucose (^18^F-FDG) positron emission tomography/computed tomography (PET/CT) is a standard imaging modality for patients with DLBLC. ^18^F-FDG PET is highly sensitive for detecting lymphoma, and plays a crucial role in disease staging and therapy monitoring, which has allowed personalized therapeutic decision making [6]. The total metabolic tumor volume (MTV) derived from baseline ^18^F-FDG PET has been shown to be associated with survival outcomes in patients with DLBCL [7,8,9,10,11,12], and novel PET imaging-derived biomarkers may further individualize the treatment of lymphoma.

Tumor heterogeneity is a pivotal prognostic factor in cancer progression, recurrence, and therapeutic resistance [13]. Moreover, tumor heterogeneity plays an important role in patient outcomes, and is correlated with tumor aggressiveness, metastasis, and molecular profiles [14,15]. Radiomic analysis can be used to assess tumor heterogeneity, and may assist with clinical outcome prognostication [16]. High-throughput radiomic features are extracted from medical images, and can reveal complex mathematical patterns in the spatial distribution of signal intensity values that are not observed visually. Radiomic analysis promotes diagnostic, predictive, and prognostic power to facilitate better clinical decision making [17]. Radiomic features have been widely explored to pursue personalized medicine in various oncology studies [18,19,20,21,22,23]; however, there is limited evidence relating to their role as prognostic factors in DLBCL [24,25].

Therefore, this study aimed to assess the prognostic value of radiomic features derived from baseline ^18^F-FDG PET in terms of survival outcomes. Moreover, we investigated the feasibility of combining clinical variables and radiomic features for the prognostic stratification of patients with DLBCL.

## 2. Materials and Methods

### 2.1. Patient Population

This study was conducted according to the Declaration of Helsinki guidelines, and approved by the Institutional Review Board and Research Ethics Committee of Hualien Tzu Chi Hospital, Buddhist Tzu Chi Medical Foundation (IRB108–251-B; 10 December 2019). The need for informed consent was waived given the retrospective nature of the study. Between September 2004 and June 2019, 83 patients with a pathological diagnosis of DLBCL who underwent pre-treatment ^18^F-FDG PET/CT were retrospectively enrolled. All patients received either R-CHOP chemotherapy or R-CVP (rituximab, cyclophosphamide, vincristine, prednisone) chemotherapy, or rituximab monotherapy in patients with a low tumor burden. Electronic charts were carefully reviewed for each patient, and data regarding patient demographics, disease characteristics, clinical course, therapy modalities, and patient outcomes were collected. All patients underwent a complete medical history, physical examination, laboratory tests, bone marrow aspiration, CT scan, and ^18^F-FDG PET/CT. The patient’s age at disease onset, Ann Arbor stage, Eastern Cooperative Oncology Group performance status, lactate dehydrogenase (LDH) level, and extranodal involvement were recorded for calculation of the IPI score [5]. Bulky disease was defined as a nodal mass larger than 10 cm in diameter.

### 2.2. Patient Follow-Up Evaluation

Initial treatment of rituximab-based chemotherapy with or without involved-field radiotherapy was conducted to the patients with DLBCL under the Clinical Practice Guidelines of the National Comprehensive Cancer Network in Oncology. Disease status was evaluated by CT or ^18^F-FDG PET/CT scan following treatment. Follow-up assessment was performed every 3 months for the first 2 years, and 6 to 12 months thereafter. The enrolled patients were followed up until disease progression or death, and these cases were counted as an event. Progression-free survival (PFS) was defined as the time from the date of diagnosis to the date of the first relapse, progression, or death from any cause. Overall survival (OS) was defined as the time from diagnosis until death from any cause [26]. Patients who did not suffer an event were censored at the date of the last known follow-up.

### 2.3. ^18^F-FDG PET/CT Scan

Patients fasted for at least 4 h before the examination and had blood glucose levels less than 150 mg/dL. Patients were injected intravenously with 5 MBq/kg of ^18^F-FDG, and PET/CT scans were performed 45 min after administration using a GE Discovery ST scanner (GE Healthcare, Milwaukee, WI, USA). PET images were acquired from the midthigh to the vertex in a static 3-dimensional mode. A CT scan without intravenous contrast medium enhancement was performed immediately prior to the PET imaging for attenuation correction. PET images were reconstructed with an ordered-subset expectation maximization algorithm (2 iterations, 21 subsets, and a 2.14-mm full width at half maximum Gaussian post-filter). The reconstructed PET image has a matrix size of 128 × 128, a pixel size of 5.47 × 5.47 mm, and a slice thickness of 3.27 mm.

### 2.4. Feature Extraction and Selection

^18^F-FDG PET images were interpreted by an expert nuclear medicine physician. To avoid interobserver variations, all images were analyzed by the same reviewer using OsiriX software (Pixmeo, Geneva, Switzerland) [27]. The results were confirmed by the other experienced nuclear medicine physician. ^18^F-FDG-avid lesions were segmented on PET images by applying the region-growing algorithm with a standardized uptake value (SUV) threshold above 2.5 for target delineation [28]. The SUV-based volumes of interest were used to compute quantitative radiomic features in PET images.

The radiomic features included 19 first-order features and 61 textural features. The first-order parameters were calculated on the basis of SUV statistics. The textural features were computed from a gray level co-occurrence matrix, gray level run length matrix (GLRLM), gray level size zone matrix (GLSZM), and neighboring gray tone difference matrix using a fixed bin width of 0.25. A total of 80 radiomic features (Supplementary Table S1) were extracted from PET images using the Pyradiomics open-source software package version 2.2.0 (Harvard Medical School, Boston, MA, USA) [29]. Radiomic features calculated by this package complies with the feature definitions described by the Imaging Biomarker Standardization Initiative (IBSI) [30,31].

To reduce dimensionality within the radiomic features, reliable features were chosen with low sensitivity to the intraclass correlation coefficient (ICC) following the literature report [32]. Subsequently, the least absolute shrinkage and selection operator (LASSO) regression algorithm [33] was employed for the chosen features. A five-fold cross-validation scheme was applied to tune the parameters of Lambda. The optimal Lambda value was identified by the minimum cross-validated criterion and the minimum criterion within one standard error. Using this method, the regression coefficients of irrelevant features were regularized to zero, and the remaining nonzero coefficients of the radiomic features were selected.

### 2.5. Statistical Analysis

The primary endpoints of this study were PFS and OS. Clinical variables and image features from the radiomic analysis were tested as potential prognostic factors. Two independent datasets were needed to build and validate the model. The data of 83 patients were randomly divided into two cohorts: 58 patients (70%) to the training dataset, and the remaining 25 patients (30%) to the validation dataset. Chi-square tests were used to compare the categorical variables between the training and validation cohorts. Receiver operating characteristic (ROC) curves were used to define the optimal cut-off values of the radiomic features by maximizing the sensitivity and specificity based on the Youden index. Cox proportional hazards regression models were used to identify the prognostic factors of PFS and OS in the training dataset. The statistically significant variables in the univariate Cox analysis were included in the stepwise multivariate Cox regression models. In both training and validation datasets, the survival curve was plotted using the Kaplan–Meier method, and the survival difference between the subgroups was assessed using a log-rank test. All statistical tests were two-sided, with a significance level of 0.05. Statistical analyses were performed using MedCalc statistical software version 19.4.1 (MedCalc Software, Ostend, Belgium) and R open-source statistical software version 3.5.2 (R Foundation, Vienna, Austria).

## 3. Results

### 3.1. Patient Characteristics

A total of 83 patients met the criteria for enrolment in the study; among whom, 65 patients were treated with the R-CHOP chemotherapy regimen, 13 with R-CVP, and 5 with rituximab monotherapy. In addition, 18 patients received involved-field radiotherapy. The median follow-up period was 41.7 months; at the time of the analysis, 35 patients (42%) suffered disease relapse or progression at a median of 9.8 months after diagnosis, and 29 patients (35%) died of the disease at a median of 10.7 months. The 5-year PFS rate was 52.3%, and the 5-year OS rate was 60.3% in the entire study population. The clinical characteristics of the patients are outlined in Table 1. No significant differences were found between the training and validation datasets (*p* = 0.111–0.755).

### 3.2. Feature Selection in the Training Cohort

The twelve radiomic features (Supplementary Table S2) with low sensitivity to the ICC (<1.10) were chosen according to the literature report [32]. These reliable features were chosen for further LASSO analysis. Based on the LASSO results (Supplementary Figure S1), MTV, gray level non-uniformity (GLN), and run length non-uniformity (RLN) both from GLRLM with nonzero regression coefficients were selected as potential prognostic factors for PFS and OS. From ROC curves, the cut-off value of MTV was 137 cm^3^, GLN_GLRLM_ was 68, and RLN_GLRLM_ was 1449. These cut-off values were used to stratify patients into those with good or poor survival outcomes.

### 3.3. Survival Analyses in the Training Cohort

The results of univariate and multivariate Cox regression analyses for the clinical variables and PET parameters are presented in Table 2 and Table 3, respectively. In the univariate analysis, the disease stage, LDH, IPI score, bulky disease of clinical variables, MTV, GLN_GLRLM_, and RLN_GLRLM_ of radiomic features were associated with PFS. Meanwhile, LDH, IPI, MTV, GLN_GLRLM_, and RLN_GLRLM_ were related to OS. These variables were entered into the multivariate Cox regression model. After multivariate analysis, RLN_GLRLM_ remained a prognostic factor for PFS, whereas the IPI and RLN_GLRLM_ maintained their prognostic significance for OS.

**Figure 1:**
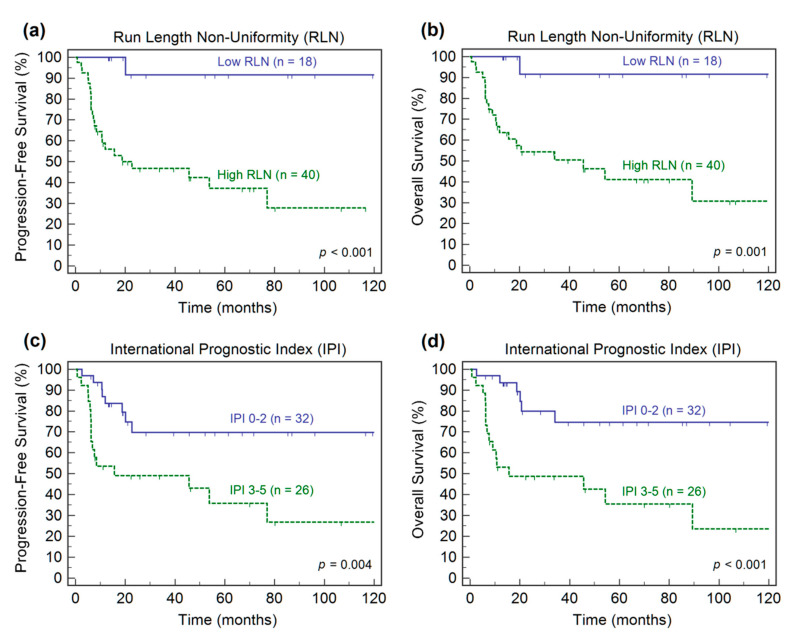
Kaplan–Meier estimates of progression-free survival and overall survival according to the baseline run length non-uniformity (**a**,**b**) and International Prognostic Index scores (**c**,**d**).

Kaplan–Meier survival analysis confirmed that the IPI score and RLN_GLRLM_ were predictive factors for both PFS and OS (Figure 1). The 5-year estimate of PFS was 35.8% in the high-risk IPI group compared to 69.8% in the low-risk IPI group. Patients with high-risk IPI scores had a 5-year OS of 35.5%, while patients with low-risk IPI scores had a 5-year OS of 74.6%. The high RLN_GLRLM_ patients had more aggressive disease, a greater risk of relapse or progression, and a lower survival rate compared to patients with low RLN_GLRLM_. Patients with a high RLN_GLRLM_ had a 5-year PFS of 37.2%, whereas patients with a low RLN_GLRLM_ had a 5-year PFS of 91.7%. Moreover, patients with a high RLN_GLRLM_ had a 5-year OS of 41.1%, whereas patients with a low RLN_GLRLM_ had a 5-year OS of 91.7%.

### 3.4. Prognostic Model Development and Validation

**Figure 2:**
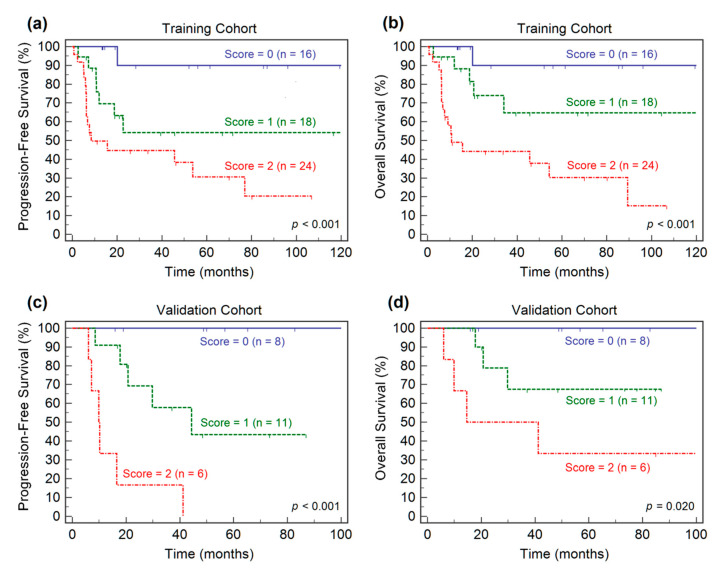
Kaplan–Meier estimates of progression-free survival and overall survival according to the prognostic stratification model in the training cohort (**a**,**b**) and the validation cohort (**c**,**d**).

A prognostic stratification model was built based on the independent risk factors presented in the multivariate Cox regression analysis for OS. The risk factors included high-risk IPI scores of the clinical variable and high RLN_GLRLM_ of the radiomic feature. A combination of the two factors, the presence or absence of each risk factor was given a score of 1 or 0, resulting in scores from 0 to 2. All patients were stratified into three risk groups: group I, with a score of 0 (none of the risk factors); group II, with a score of 1 (one risk factors); and group III, with a score of 2 (two risk factors). In the training dataset, Kaplan–Meier analyses of PFS and OS demonstrated the ability of the prognostic stratification model (Figure 2a,b). Survival curves revealed that the three risk groups were significantly different with regard to PFS and OS. The 5-year PFS of patients in groups I to III were 90.0%, 54.2%, 30.6% (*p* < 0.001), respectively, and the 5-year OS were 90.0%, 64.7.0%, 30.3% (*p* < 0.001), respectively.

In the validation dataset, survival curves generated through Kaplan–Meier analysis indicated that the prognostic stratification model identified three risk groups for survival outcomes (Figure 2c,d). The patients in group I had significantly higher 5-year PFS (100% vs. 43.3% vs. 0%, *p* < 0.001) and OS (100% vs. 67.5% vs. 33.3%, *p* = 0.020) rates than those in groups II and III.

## 4. Discussion

The present study investigated the use of radiomic analysis of ^18^F-FDG PET for predicting survival outcomes in patients with DLBCL. Our results demonstrate that baseline ^18^F-FDG PET radiomics have prognostic value, and that RLN_GLRLM_ is an independent prognostic factor for both PFS and OS. The RLN_GLRLM_ provides a way of featuring for tumor heterogeneity, driven by the genomic diversity that enables the tumor to evolve and adapt to anticancer treatments [15,34]. Therefore, it can be reasoned that the assessment of tumor heterogeneity allows us to anticipate patient outcomes. Moreover, a prognostic stratification model was devised to identify the risk groups of patients based on integrating clinical and imaging prognostic factors. The proposed model showed the complementary roles of combining clinical information with tumor heterogeneity and allowed the stratification of three risk groups according to survival outcomes in patients with DLBCL.

Many PET radiomic features are currently under investigational use, and different studies have reported different radiomic features for predicting the survival outcome of lymphoma [35,36,37,38,39]. To keep the data dimensionally low to avoid overfitting, only 12 radiomic features with low ICC sensitivity were evaluated for clinical endpoints in this study. The cohort was split into a training dataset (70%) and an internal validation dataset (30%). A LASSO algorithm was further used for feature selection in order to achieve the best accuracy for PFS and OS prognostication. The radiomic feature identified in the study, RLN_GLRLM_, was a valuable imaging biomarker after multivariable analyses. RLN_GLRLM_ estimates the similarity of run lengths throughout the image, where a lower value indicates higher homogeneity. A higher RLN_GLRLM_ was associated with a worse prognosis, suggesting that the measurement of tumor heterogeneity of ^18^F-FDG PET distribution is an essential biomarker in patients with DLBCL.

The literature on molecular imaging radiomics for DLBCL is limited. A few studies have been conducted to investigate the usefulness of PET radiomic features in determining the survival in DLBCL. Parvez et al. [38] found that GLN_GLSZM_ correlated with disease-free survival, and that kurtosis correlated with OS. Moreover, Aide et al. [35,40] found that skewness of skeletal heterogeneity was a prognostic factor for PFS, and long-zone high gray level emphasis from GLSZM was a prognostic parameter for 2-year event-free survival. Recently, Cottereau et al. [41] reported that the radiomic feature characterizing lesion dissemination was associated with PFS and OS. Our findings are in line with those of studies indicating that the PET-derived radiomic features are useful for patient outcome prognostication in DLBCL. Previous studies have indicated that MTV can be used to determine the prognosis of patients with DLBCL [7,8,9,10,11,12]. Our results are not in contradiction with those of the studies. In univariate analysis, MTV demonstrated prognostic significance; however, in multivariate analysis, MTV did not correlate with PFS and OS, presumably due to the small sample size. In lymphoma, few reports have indicated that the performance of PET metabolic parameters for survival prognostication is poor compared to that of PET radiomic features [42,43]. On the contrary, Wang et al. [39] reported that radiomics are not superior to traditional imaging parameters. Notwithstanding, our data suggest that features of tumor heterogeneity may serve as a complementary indicator of MTV. Further external validation is required in a larger cohort population to validate our findings.

Tumor heterogeneity has the potential to impact the prognosis of patients with DLBCL [44]. Lymphoma is a system malignancy, which lacks a primary tumor in the majority of cases. A biopsy is generally performed for a single lesion site in routine clinical practice. Thus, it might be more relevant to explore the tumor heterogeneity across the entire tumor volume than with a single site biopsy in DLBCL. In this study, a tumor heterogeneity feature from the entire tumor volume was combined with the clinical IPI to construct a prognostic stratification model. Our findings highlight the benefit of an integrated approach that includes IPI and radiomics for evaluating patients with DLBCL at initial diagnosis. Currently, a qualitative assessment of response using ^18^F-FDG PET has been implemented into the clinical management of DLBCL. However, patients with DLBCL failed to achieve significant survival improvement after the qualitative ^18^F-FDG PET response directed-treatment strategy [45]. Radiomics provides a more sophisticated quantitative measure of ^18^F-FDG PET. We further combined radiomics with the clinical IPI system into a survival prediction model. Because radiomics portrays tumor heterogeneity, which is different from the clinical information provided by the IPI score, these two features may have complementary roles. A combination of the two risk factors may more comprehensively depict the survival risk of DLBCL. Future clinical trials are warranted to test the ability of our proposed model to guide tailored treatment strategies.

Despite the usefulness of radiomics, it does have certain limitations. First, radiomics are extracted in terms of MTV, and the method of MTV measurement is inconsistent among different working groups. A recent report [28] indicated that different methods predicted prognosis, but those with a SUV ≥ 2.5 had the best interobserver agreement and were easiest to apply in DLBCL; this was the method we selected in the current study. Moreover, the threshold used to divide patients into high- and low-risk groups depends on the method of MTV measurement. Thus, setting of common criteria for standardization of the MTV calculation is warranted [46]. Second, the SUV discretization step in computing textural features can influence repeatability [47]. In our work, a reliable discretization using a fixed size of bins was adopted, which was shown to be more appropriate in clinical cases [48]. However, the optimal bin size value could not be identified (i.e., the extraction of reliable radiomic features has not been thoroughly investigated). Further investigation of the optimal size of bins for survival prognostication should be considered. Third, the reliability of radiomic features and their ability to predict clinical outcomes is highly dependent on the choice of feature extraction platform [49]. Future radiomic studies should still ensure platforms are IBSI-compliant, as was the platform that we adopted in the current study. Finally, radiomic features can be sensitive to the imaging acquisition and reconstruction settings [50]. Therefore, a radiomic-based model might not be directly applied to different imaging centers, which limits its usefulness in clinical practice. Further research is necessary to validate our findings using a post-reconstruction harmonization [51] approach in multicenter trials.

We acknowledge that our research is exploratory and that there are several limitations. Like most radiomic studies, selection bias could not be avoided due to the retrospective nature of the study. Furthermore, since our analysis was based on a small number of patients, the lack of statistically significant differences should be interpreted with caution, as a statistical difference may be evident with a larger population. Besides, the interobserver variability could be affected by different image readers. In addition, current molecular genetic studies have identified DLBCL subtypes with less favorable survival outcomes, such as the activated B-cell subtype or MYC oncogene rearrangement [11,52]. However, only 12 patients in our cohort underwent subtyping. Whether the radiomic features derived from ^18^F-FDG PET are associated with the different subtypes of DLBCL requires further investigation. Finally, the rituximab-based regimens and the radiotherapy doses varied throughout the study. This study demonstrated that the identified radiomic feature has prognostic value in DLBCL, but the underlying biological meaning remains to be further explored in larger, multi-institutional cohorts before they can be applied to clinical decision making.

## 5. Conclusions

Our results indicate that the baseline ^18^F-FDG PET radiomic feature, RLN_GLRLM_, serves as an independent prognostic factor for survival outcomes. Furthermore, a prognostic stratification model combining the IPI and RLN_GLRLM_ can be useful for risk stratification of patients with DLBCL. Our findings may be clinically helpful in guiding personalized therapeutic strategies.

## Figures and Tables

**Table 1:** Clinical Characteristics of Patients in the Training and Validation Cohorts.

Characteristic	Overall (*n* = 83)	Training (*n* = 58)	Validation (*n* = 25)	*p*-Value
Sex				
Female	32 (39%)	23 (40%)	9 (36%)	0.755
Male	51 (61%)	35 (60%)	16 (64%)	
Age, median (range), years	61 (19–86)	61 (19-86)	59 (19–81)	0.550
Ann Arbor stage				
Early (I–II)	33 (40%)	25 (43%)	8 (32%)	0.345
Advanced (III–IV)	50 (60%)	33 (57%)	17 (68%)	
ECOG performance status				
0/1	59 (71%)	41 (71%)	18 (72%)	0.904
2–4	24 (29%)	17 (29%)	7 (28%)	
LDH				
Normal	23 (28%)	17 (29%)	6 (24%)	0.622
Elevated (>271 U/L)	60 (72%)	41 (71%)	19 (76%)	
Extranodal sites				
No	49 (59%)	35 (60%)	14 (56%)	0.713
Yes	34 (41%)	23 (40%)	11 (44%)	
IPI score				
Low-risk (0–2)	41 (49%)	32 (55%)	9 (36%)	0.111
High-risk (3–5)	42 (51%)	26 (45%)	16 (64%)	
Bulky disease (>10 cm)	9 (11%)	7 (12%)	2 (11%)	0.587
R-CHOP	65 (78%)	47 (81%)	18 (72%)	0.362
Radiotherapy	18 (22%)	12 (21%)	6 (24%)	0.739

ECOG, Eastern Cooperative Oncology Group; LDH, lactate dehydrogenase; IPI, International Prognostic Index; R-CHOP, rituximab-cyclophosphamide, hydroxydaunorubicin, oncovin, prednisolone chemotherapy.

**Table 2:** Univariate and Multivariate Analyses for Prognostic Factors of Progression-free Survival.

	Univariate Analysis		Multivariate Analysis	
	HR (95% CI)	*p*-Value	HR (95% CI)	*p*-Value
Clinical variables				
Age (>60 years)	2.012 (0.876–4.618)	0.098		
Female vs. Male	1.178 (0.515–2.695)	0.697		
Stage (I–II vs. III–IV)	2.618 (1.035–6.621)	0.042 *		0.980
ECOG (0/1 vs. 2–4)	1.931 (0.819–4.553)	0.132		
LDH (≤271 vs. >271 U/L)	3.151 (1.248–7.958)	0.015 *		0.748
Extranodal sites (no vs. yes)	1.725 (0.774–3.845)	0.182		
IPI score (0–2 vs. 3–5)	3.248 (1.386–7.608)	0.006 *		0.224
Bulky disease (>10 cm)	3.179 (1.147–8.812)	0.026 *		0.282
PET parameters				
MTV (>137 cm^3^)	13.64 (1.837–101.2)	0.011 *		0.169
GLN_GLRLM_ (>68)	15.42 (2.078–114.3)	0.007 *		0.155
RLN_GLRLM_ (>1449)	15.66 (2.107–116.5)	0.007 *	15.66 (2.107–116.5)	0.007 *

HR, hazard ratio; CI, confidence interval; ECOG, Eastern Cooperative Oncology Group; LDH, lactate dehydrogenase; IPI, International Prognostic Index; MTV, metabolic tumor volume; GLN, gray level non-uniformity; GLRLM, gray level run length matrix; RLN, run length non-uniformity; *, statistically significant.

**Table 3:** Univariate and Multivariate Analyses for Prognostic Factors of Overall Survival.

	Univariate Analysis		Multivariate Analysis	
	HR (95% CI)	*p*-Value	HR (95% CI)	*p*-Value
Clinical variables				
Age (>60 years)	2.301 (0.958–5.520)	0.062		
Female vs. Male	1.286 (0.538–3.072)	0.571		
Stage (I–II vs. III–IV)	2.658 (0.974–7.253)	0.056		
ECOG (0/1 vs. 2–4)	2.278 (0.944–5.495)	0.066		
LDH (≤271 vs. >271 U/L)	3.270 (1.205–8.875)	0.020 *		0.620
Extranodal sites (no vs. yes)	2.137 (0.921–4.957)	0.077		
IPI score (0–2 vs. 3–5)	4.393 (1.714–11.26)	0.002 *	2.626 (1.001–6.885)	0.049 *
Bulky disease (>10 cm)	1.819 (0.611–5.408)	0.282		
PET parameters				
MTV (>137 cm^3^)	11.45 (1.538–85.19)	0.017 *		0.343
GLN_GLRLM_ (>68)	13.06 (1.755–97.20)	0.012 *		0.215
RLN_GLRLM_ (>1449)	13.19 (1.771–98.26)	0.011 *	8.636 (1.104–67.57)	0.040 *

HR, hazard ratio; CI, confidence interval; ECOG, Eastern Cooperative Oncology Group; LDH, lactate dehydrogenase; IPI, International Prognostic Index; MTV, metabolic tumor volume; GLN, gray level non-uniformity; GLRLM, gray level run length matrix; RLN, run length non-uniformity; *, statistically significant.

## Data Availability

The data presented in this study are available on request from the corresponding author. The data are not publicly available due to the privacy and ethical restrictions.

## Supplementary Materials

Supplementary materials can be found at https://www.mdpi.com/2075-4418/11/1/36/s1.
