# Supplementary material for: Prognostic Value of Baseline Radiomic Features of 18F-FDG PET in Patients with Diffuse Large B-Cell Lymphoma
Source: Diagnostics (Basel). 2020 Dec 28;11(1):36. doi: 10.3390/diagnostics11010036 (PMC7824203; doi:10.3390/diagnostics11010036)
Supplement: Supplementary file 1 [file diagnostics-11-00036-s001.pdf]

Supplementary Material

Prognostic Value of Baseline Radiomic Features of ^{18}F-FDG PET in Patients with Diffuse Large B-cell Lymphoma

Kun-Han Lue [1], Yi-Feng Wu [2,3], Hsin-Hon Lin [4,5,6], Tsung-Cheng Hsieh [7], Shu-Hsin Liu [1,8], Sheng-Chieh Chan [3,8] and Yu-Hung Chen [3,8,*]

1 Department of Medical Imaging and Radiological Sciences, Tzu Chi University of Science and Technology, Hualien 97005, Taiwan; john.lue@protonmail.com
2 Department of Hematology and Oncology, Hualien Tzu Chi Hospital, Buddhist Tzu Chi Medical Foundation, Hualien 97004, Taiwan; wuyifeng43@gmail.com
3 Department of Medicine, College of Medicine, Tzu Chi University, Hualien 97004, Taiwan
4 Medical Physics Research Center, Institute for Radiological Research, Chang Gung University/Chang Gung Memorial Hospital, Taoyuan 33302, Taiwan; muska0345@outlook.com
5 Department of Radiation Oncology, Chang Gung Memorial Hospital, Taoyuan 33305, Taiwan
6 Department of Nuclear Medicine, Keelung Chang Gung Memorial Hospital, Keelung 20401, Taiwan
7 Institute of Medical Sciences, Tzu Chi University, Hualien 97004, Taiwan; tchsieh@gms.tcu.edu.tw
8 Department of Nuclear Medicine, Hualien Tzu Chi Hospital, Buddhist Tzu Chi Medical Foundation, Hualien 97004, Taiwan; kaopectin@yahoo.com.tw (S.-H.L.); williamsm.tw@gmail.com (S.-C.C.)
* Correspondence: jedimasterchen@hotmail.com; Tel.: +886-3-856-1825

Table S1. List of ^{18}F-FDG PET Radiomic Features

**Classes**	**Feature Name**
First Order Voxel Statistics	10th percentile
	90th percentile
	Energy
	Entropy
	Interquartile Range
	Kurtosis
	Maximum
	Mean
	Absolute Deviation
	Median
	Minimum
	Range
	Robust Mean Absolute Deviation
	Root Mean Squared
	Skewness

	Total Energy
	Uniformity
	Variance
	Metabolic Tumor Volume
Gray Level Co-occurrence Matrix (GLCM)	Autocorrelation
	Cluster Prominence
	Cluster Shade
	Cluster Tendency
	Contrast
	Correlation
	Difference Average
	Difference Entropy
	Difference Variance
	Inverse Difference
	Inverse Difference Moment
	Inverse Difference Moment Normalized
	Inverse Difference Normalized
	Informational Measure of Correlation 1
	Informational Measure of Correlation 2
	Inverse Variance
	Joint Average
	Joint Energy
	Joint Entropy
	Maximal Correlation Coefficient
	Maximum Probability
	Sum Average
	Sum Entropy
	Sum Squares
Gray Level Run Length Matrix (GLRLM)	Gray Level Non-Uniformity
	Gray Level Non-Uniformity Normalized
	Gray Level Variance
	High Gray Leve Run Emphasis
	Long Run Emphasis
	Long Run High Gray Level Emphasis
	Long Run Low Gray Level Emphasis
	Low Gray Level Run Emphasis
	Run Entropy

	Run Length Non-Uniformity
	Run Length Non-Uniformity Normalized
	Run Percentage
	Run Variance
	Short Run Emphasis
	Short Run High Gray Level Emphasis
	Short Run Low Gray Level Emphasis
Gray Level Size Zone Matrix (GLSZM)	Gray Level Non-Uniformity
	Gray Level Non-Uniformity Normalized
	Gray Level Variance
	High Gray Level Zone Emphasis
	Large Area Emphasis
	Large Area High Gray Level Emphasis
	Large Area Low Gray Level Emphasis
	Low Gray Level Zone Emphasis
	Size Zone Non-Uniformity
	Size Zone Non-Uniformity Normalized
	Small Area Emphasis
	Small Area High Gray Level Emphasis
	Small Area Low Gray Level Emphasis
	Zone Entropy
	Zone Percentage
	Zone Variance
Neighboring Gray Tone Difference Matrix (NGTDM)	Busyness
	Coarseness
	Complexity
	Contrast
	Strength

The future explanations are available at thttps://pyradiomics.readthedocs.io/

Table S2. List of Chosen Radiomic Features

Classes	Feature Name
First Order Voxel Statistics	Metabolic Tumor Volume
Gray Level Co-occurrence Matrix (GLCM)	Cluster Prominence
	Cluster Tendency
	Inverse Difference
	Inverse Difference Moment
	Inverse Variance
	Sum Squares
Gray Level Run Length Matrix (GLRLM)	Gray Level Non-Uniformity
	Long Run High Gray Level Emphasis
	Run Length Non-Uniformity
	Run Percentage
	Short Run Emphasis

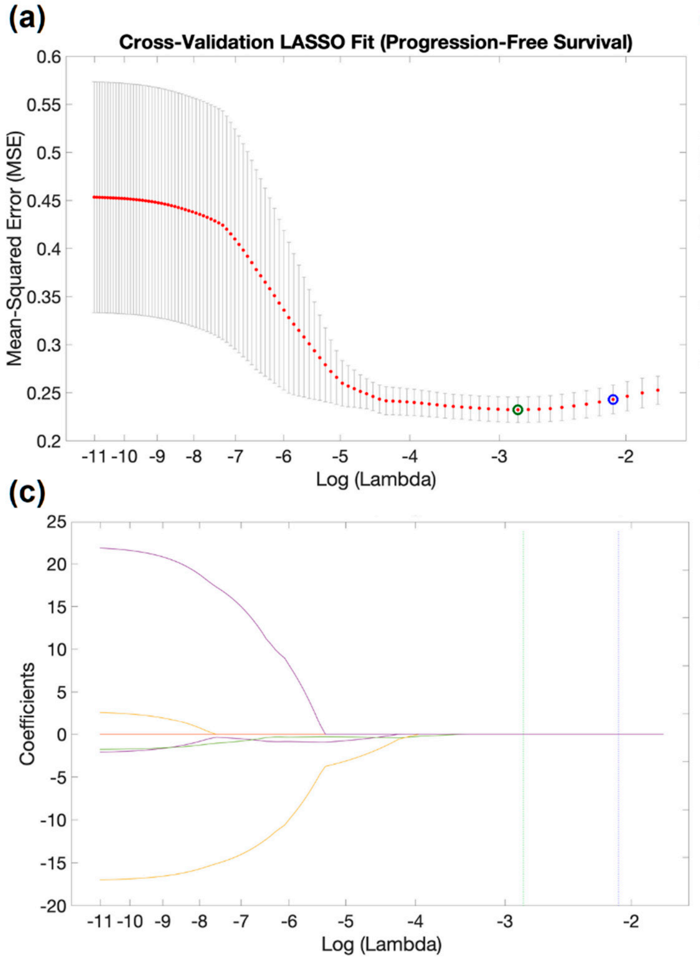

Figure S1. Radiomic feature selection using the Least Absolute Shrinkage and Selection Operator (LASSO) regression with five-fold cross-validation. The optimal Lambda value was identified by the minimum mean-squared error (MSE) and by the minimum MSE within one standard error. Feature selection and coefficient profiles for the prediction of progression-free survival (**a**, **c**) and overall survival (**b**, **d**).
